# Xenon inhibits excitatory but not inhibitory transmission in rat spinal cord dorsal horn neurons

**DOI:** 10.1186/1744-8069-6-25

**Published:** 2010-05-05

**Authors:** Stefan K Georgiev, Hidemasa Furue, Hiroshi Baba, Tatsuro Kohno

**Affiliations:** 1 Division of Anaesthesiology, Niigata University Graduate School of Medical and Dental Sciences, 1-757 Asahimachi, Chuo ku, Niigata 951-8510, Japan; 2 Department of Integrative Physiology, Graduate School of Medical Sciences, Kyushu University, Fukuoka 812-8582, Japan; 3 Department of Information Physiology, National Institute for Physiological Sciences, Myodaiji 5-1, Okazaki 444-8787, Japan; 4 Pain Mechanism Research Group, 1-757 Asahimachi, Chuo ku, Niigata 951-8510, Japan

## Abstract

**Background:**

The molecular targets for the promising gaseous anaesthetic xenon are still under investigation. Most studies identify *N*-methyl-D-aspartate (NMDA) receptors as the primary molecular target for xenon, but the role of α-amino-3-hydroxy-5-methyl-4-isoxazole-4-propionic acid (AMPA) receptors is less clear. In this study we evaluated the effect of xenon on excitatory and inhibitory synaptic transmission in the superficial dorsal horn of the spinal cord using *in vitro *patch-clamp recordings from rat spinal cord slices. We further evaluated the effects of xenon on innocuous and noxious stimuli using *in vivo *patch-clamp method.

**Results:**

*In vitro*, xenon decreased the amplitude and area under the curve of currents induced by exogenous NMDA and AMPA and inhibited dorsal root stimulation-evoked excitatory postsynaptic currents. Xenon decreased the amplitude, but not the frequency, of miniature excitatory postsynaptic currents. There was no discernible effect on miniature or evoked inhibitory postsynaptic currents or on the current induced by inhibitory neurotransmitters. *In vivo*, xenon inhibited responses to tactile and painful stimuli even in the presence of NMDA receptor antagonist.

**Conclusions:**

Xenon inhibits glutamatergic excitatory transmission in the superficial dorsal horn *via *a postsynaptic mechanism. There is no substantial effect on inhibitory synaptic transmission at the concentration we used. The blunting of excitation in the dorsal horn lamina II neurons could underlie the analgesic effect of xenon.

## Background

Xenon has an excellent anaesthetic profile and if used with an elaborate low-flow delivery system, xenon has the potential to become a main-line anaesthetic [1-3], replacing nitrous oxide in balanced anaesthesia or it could even be used as a mono-anaesthetic. Accumulating reports on xenon's organ-protective properties [4,5] suggest it may have promising use in high-risk patients. Significant progress has been made toward elucidating how xenon produces anaesthesia and several potential molecular targets have been identified. Current evidence strongly indicates that xenon inhibits excitatory glutamatergic signalling, but it is unclear which receptor subtype is involved in its anaesthetic action [6]. Most studies agree on *N*-methyl-D-aspartate (NMDA) receptor inhibition [7,8], but reports on xenon's effects on α-amino-3-hydroxy-5-methyl-4-isoxazole-4-propionic acid (AMPA) receptors are contradictory [9,10]. A recent study using murine brain slices reported inhibition of both NMDA and AMPA receptors in amygdala neurons, a brain region related to anaesthetic-induced amnesia, modulation of pain perception and emotions [11]. However, pain processing is not confined to a single brain region, which makes us think it is appropriate to investigate the effect of xenon at the gate, that is, the superficial dorsal horn of the spinal cord. The spinal cord was proposed as site of the analgesic effect of xenon when neuronal responses to touch and pinch, obtained by extracellular recordings, were suppressed by the inert gas in spinal cord intact [12] and transected [13] cats, but this hypothesis was not further investigated in detail. Spinal cord slice preparations with attached dorsal roots show preserved local neuronal networks and afferent inputs, and thus, have proved useful in analyzing the mechanisms of pain transmission and drug pharmacology. The aim of the present study was to characterize the effects of xenon on excitatory and inhibitory synaptic transmission in dorsal horn lamina II (*substantia gelatinosa*, SG), the first processing centre in the nociceptive information flow.

## Results

### Effects of xenon on excitatory synaptic transmission revealed by *in vitro *patch-clamp recordings

Xenon did not directly affect postsynaptic membrane properties, because the amount of holding current required to maintain the neurons at -70 or 0 mV (% change = 3.9 ± 0.3; n = 55) and the resting membrane potential (control, -65.1 ± 0.7 mV vs. xenon, -65.3 ± 0.8 mV, n = 15, P = 0.7) were unaffected by xenon.

**Figure 1:**
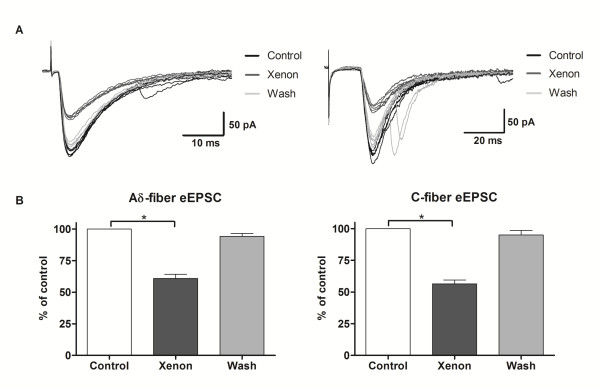
**Effect of xenon on eEPSCs**. A - Excitatory post synaptic currents evoked at holding potential (HP) = -70 mV by primary afferents electrical stimulation recruiting Aδ- (left) and C-fibres (right) before (black), during (dark grey) and after (light grey) xenon application. Five consecutive traces are shown for each modality. Note the decrease in amplitude during xenon application. B - Xenon reversibly decreased the mean amplitude of monosynaptic currents evoked by Aδ- (left, n = 10) and C- (right, n = 9) afferent fibre stimulation.

We first investigated the effects of xenon on evoked excitatory postsynaptic currents (eEPSCs). Electrical stimulation of the dorsal root evoked inward synaptic currents at -70 mV. These currents are believed to be mediated exclusively by AMPA receptors, because they were almost completely blocked by 6-cyano-7-nitroquinoxaline-2,3-dione (CNQX, 10 μM, current amplitude = 4.0 ± 0.5% of control, n = 4, data not shown). Only data meeting the criteria for monosynaptic currents were further analyzed. Xenon decreased the amplitude of Aδ-fibre intensity stimulation-evoked current to 60 ± 3% of control (n = 10, P < 0.01; Fig. 1A, B) and the integrated area to 59 ± 4% of control (n = 10, P < 0.01; data not shown). The effect of xenon was typically noticeable within 2-3 minutes following xenon application, reached its maximum by 3 minutes and disappeared approximately 3 minutes after xenon's termination. Xenon similarly decreased the current amplitude to 57 ± 3% of the control level (n = 9, P < 0.01; Fig. 1A, C) and the integrated area to 58 ± 3% (n = 9, P < 0.01; data not shown) for C-fibre mediated responses in all tested neurons.

**Figure 2:**
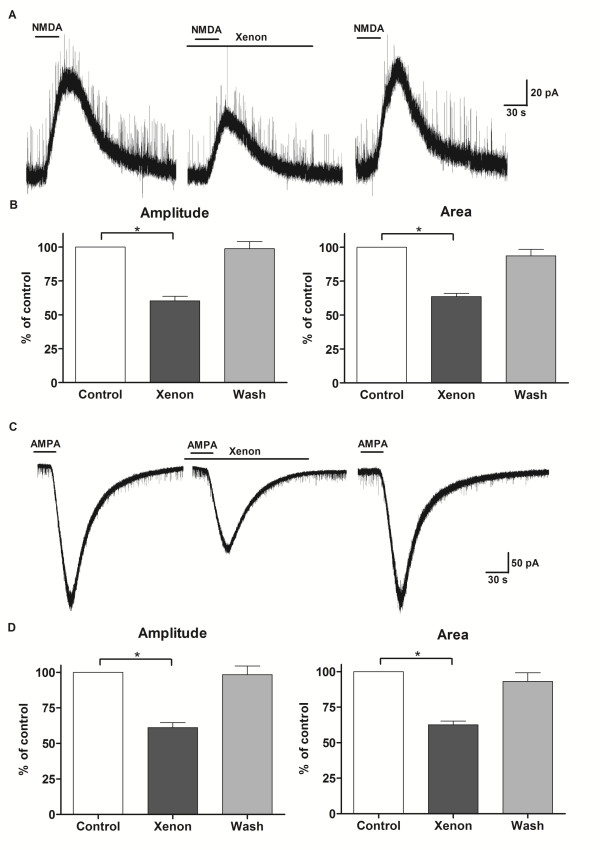
**Exogenous agonist-induced currents inhibited by xenon**. A - Representative traces showing NMDA (50 μM, 30 s)-induced outward currents at HP = +40 mV, which were reversibly reduced by xenon (50%). Drug application is marked with bars. B - Xenon decreased the amplitude and integrated area of NMDA-induced currents in lamina II neurons (n = 8). *P < 0.05 compared with control. C - AMPA (10 μM, 30 s)-induced inward currents at HP = -70 mV, which were reversibly reduced by xenon (50%). D - Xenon decreased the amplitude and integrated area of NMDA-induced currents in lamina II neurons (n = 7). *P < 0.05 compared with control.

Exogenous agonist-induced currents are representative for activation of postsynaptic receptors. Exogenous application of NMDA (50 μM, 30 s) elicited an outward current at +40 mV (Fig. 2A). In the presence of xenon, but not nitrogen, the mean amplitude of NMDA-induced current was reversibly reduced to 60 ± 3% (n = 8, P < 0.01; Fig. 2A, B) and the integrated area to 63 ± 2% (n = 8, P < 0.01; Fig. 2B) of control values. Bath-applied AMPA (10 μM, 30 s) induced an inward current at -70 mV. During xenon application the peak amplitude and integrated area of AMPA-induced current decreased to 61 ± 3% (n = 7, P < 0.01) and 63 ± 2% (n = 7, P < 0.01; Fig. 2C, D) of the control values, respectively, and returned to initial values after washout.

**Figure 3:**
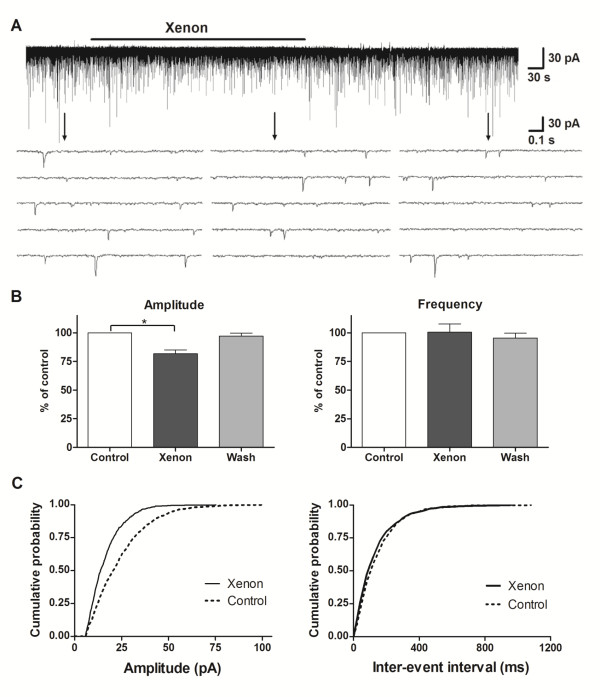
**Xenon decreased mEPSC amplitude, but not mEPSC frequency**. A - Two-scale traces of mEPSC recorded at HP = -70 mV in the presence of tetrodotoxin (1 μM) before, during and after xenon application. B - The effect of xenon on miniature excitatory postsynaptic current (mEPSC) amplitude (n = 16) and frequency (n = 16). *P < 0.05 compared with control. C - Cumulative histograms of mEPSC amplitude (left) and inter-event intervals (right) from a representative neuron showing the shift to the left of mEPSC amplitude under xenon exposure. Control trace was calculated from 470 events and xenon trace was calculated from 490 events.

Miniature excitatory postsynaptic currents (mEPSC) were isolated by adding tetrodotoxin (TTX, 1 μM) to the perfusing solution. In the presence of xenon, the mean mEPSC amplitude decreased to 81 ± 5% of control (n = 16, P < 0.01; Fig. 3A, B). In 12 of 16 neurons tested, the cumulative histograms of amplitude distribution were significantly shifted to the left after perfusion with xenon (Fig. 3C). The mean relative mEPSC frequency remained unchanged (100 ± 6% of control, n = 16, P = 0.2; Fig. 3A-C), as did the decay time constant (100 ± 2% of control, n = 16, P = 0.8, data not shown). When the cumulative histograms of the inter-event intervals were analyzed by Kolmogorov-Smirnov test, there was an increase in inter-event intervals in two neurons and a decrease in three, but in the majority (11/16) of neurons, the inter-event intervals were not affected. Xenon-induced changes in mEPSC amplitude and frequency usually disappeared within 3-4 minutes after discontinuation of the drug.

### Effects of xenon on excitatory synaptic transmission revealed by *in vivo *patch-clamp recordings

**Figure 4:**
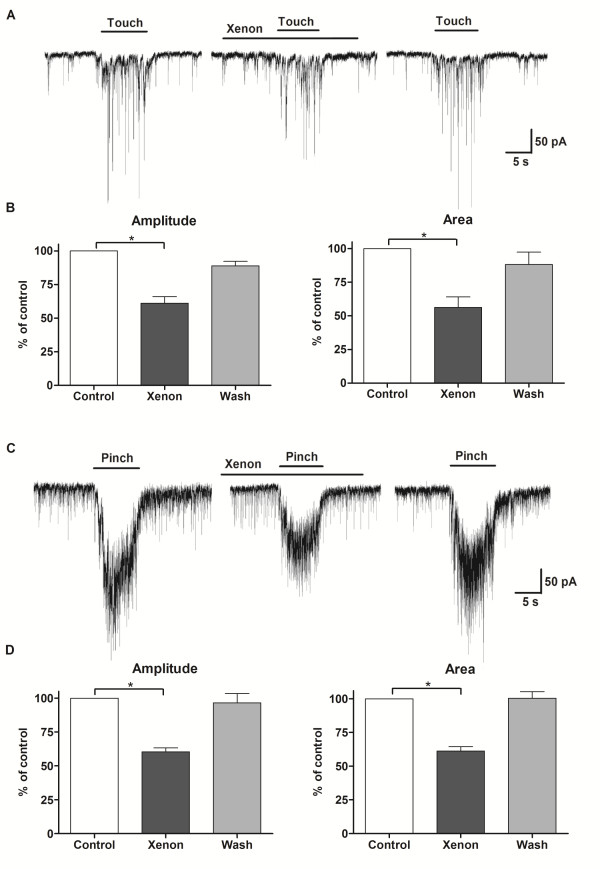
**Xenon inhibited the responses to touch and pinch *in vivo***. A - Representative traces showing the decrease in the response to light touch of the hind limb during xenon application (HP = -70 mV) recorded using *in vivo *patch-clamp method. B - Diagram showing the reversible decrease in mean amplitude and integrated area of the touch response during xenon application (n = 4). *P < 0.05 compared with control. C - Response to skin fold pinch during xenon application. D - The mean amplitude and integrated area of pinch-evoked EPSCs decreased in the presence of xenon (n = 6). n is the number of neurons.

We further looked to confirm the *in vitro*-obtained data with *in vivo *recordings. Light brushing or pinching with forceps of a specific area of the hind limb produced a barrage of EPSCs, which persisted during the whole stimulus and terminated with its termination (Fig. 4A, C). Both mean amplitude (61 ± 4% of control, n = 4, P < 0.01; Fig. 4B) and integrated area (56 ± 6% of control, n = 4, P < 0.01; Fig. 4B) of touch-evoked responses were inhibited by xenon. Amplitude (60 ± 3% of control, n = 6, P < 0.01; Fig. 4D) and integrated area (61 ± 3% of control, n = 6, P < 0.01; Fig. 4D) of pinch-evoked responses were similarly inhibited by xenon. We further investigated the nature of these responses by applying the NMDA receptor antagonist D-2-amino-5-phosphonovaleric acid (APV, 50 μM) prior and during xenon application. In the presence of APV, xenon similarly inhibited the responses to touch (amplitude: 64 ± 2% of control, n = 3, P < 0.01; integrated area: 62 ± 2% of control, n = 3, P < 0.01, see additional file 1) and pinch (amplitude: 65 ± 3% of control, n = 4, P < 0.01; integrated area: 61 ± 3% of control, n = 4, P < 0.01; see additional file 2) stimuli. The spontaneous EPSCs as well as touch (amplitude: 5 ± 1% of control, n = 2; integrated area: 1 ± 1% of control, n = 2) and pinch (amplitude: 6 ± 1% of control, n = 2; integrated area: 3 ± 1% of control, n = 2) stimulation-evoked EPSCs almost completely disappeared in the presence of CNQX (20 μM).

### Effects of xenon on inhibitory synaptic transmission revealed by *in vitro *patch-clamp recordings

**Figure 5:**
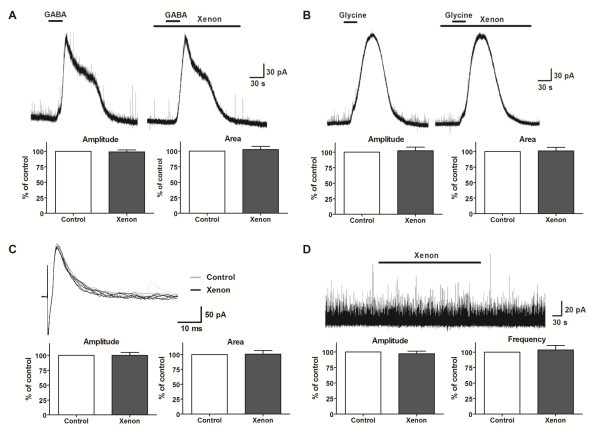
**Xenon did not affect inhibitory synaptic transmission in lamina II neurons**. Representative traces and diagrams showing no change in amplitude and integrated area of the currents induced by exogenous GABA (A) or glycine (B) in the presence of xenon. C - Focal stimulation induced inhibitory postsynaptic currents before (grey) and during (black) xenon application. Neither amplitude nor integrated area changed in the presence of xenon. D - Trace and diagram showing the lack of an effect of xenon on the mean amplitude or frequency of miniature inhibitory postsynaptic currents.

The next step was to examine whether xenon modulates inhibitory synaptic transmission in SG neurons at a holding potential of 0 mV. The amplitude of the current elicited by exogenous application of GABA (1 mM, 30 s) was not affected by xenon (99 ± 3% of control, n = 8, P = 0.7; Fig. 5A), and nor was the integrated area (102 ± 5% of control, n = 8, P = 0.6; Fig. 5A). Glycine (1 mM, 30 s)-induced current amplitude (102 ± 5% of control, n = 8, P = 0.7; Fig. 5B) and integrated area (101 ± 5% of control, n = 8, P = 0.9; Fig. 5B) were also not affected by xenon. Focal stimulation near the recorded neuron elicited monosynaptic inhibitory postsynaptic currents (IPSCs). The peak amplitude (100 ± 4% of control, n = 6, P = 0.9; Fig. 5C), integrated area (101 ± 5% of control, n = 6, P = 0.8; Fig. 5C) and decay time (99 ± 3% of control, n = 6, P = 0.6, not shown) did not change in the presence of xenon. The above evoked IPSC are known to be mediated by GABA and glycine receptors, but since no effect was observed on the compound eIPSCs, there were no further studies done. Adding TTX (1 μM) to the Krebs solution allowed for recording of mIPSCs. During xenon application, the mIPSC mean amplitude was 97 ± 4% of the control level (n = 9, P = 0.8; Fig. 5D), mean frequency was 102 ± 6% of the control level (n = 9, P = 0.8; Fig. 5D), and decay time was 101 ± 2% of the control level (n = 9, P = 0.9, not shown). Analysis of cumulative distribution histograms showed a decrease in mIPSC amplitude in one neuron, increase in another and no change in seven. Inter-event intervals decreased in two neurons and increased in one, but remained unchanged in the majority (6/9) of tested cells.

## Discussion

In this study we investigated the effects of xenon on excitatory and inhibitory synaptic transmission in the superficial dorsal horn using *in vitro *and *in vivo *patch-clamp methods. Xenon inhibited excitatory synaptic transmission by postsynaptic inhibition of both NMDA and AMPA receptors, but had no effect on inhibitory synaptic transmission in adult rat dorsal horn neurons.

We used 50% xenon in our experiments for technical and financial reasons. Because the minimal alveolar concentration (MAC) of xenon is estimated to be 63-71% in humans and between 86% [14] and 161% [15] in rats, the concentration we used (50% xenon) is 0.31-0.58 rat MAC. Although this concentration is insufficient to induce anaesthesia, xenon is known to have analgesic properties even at sub-anaesthetic (0.3 MAC) concentrations in humans [16,17] and in rats [18], so we believe that this xenon concentration is sufficient to modulate nociceptive synaptic transmission at the dorsal horn level.

The role of ionotropic glutamatergic receptors as anaesthetic targets has emerged during the last decade and NMDA receptors have had a central place in most reports. Several studies have demonstrated that xenon inhibits NMDA receptors in various non-neuronal [19] and neuronal [7,9] preparations. Our results confirm the inhibitory action of xenon on NMDA receptors and extend this to SG neurons. The inhibition of NMDA receptor-mediated signalling in dorsal horn neurons could account for the antinociceptive action and could indirectly, through decreasing the sensory input to the ventral horn, contribute to the immobilizing action of xenon. Additionally, NMDA receptor antagonism by xenon could prevent plasticity-related phenomena like central sensitization or excitotoxicity. Actually, xenon has prevented the development of long-term potentiation [20], presumably by inhibiting NMDA or/and AMPA signalling in the superficial dorsal horn. An fMRI study demonstrated that sub-anaesthetic doses of xenon suppressed the enhanced responsiveness in pain-related brain areas induced by repeated painful stimulation [21]. In another study, xenon protected against neuronal injury in an NMDA excitotoxicity model [22]. Future investigations should determine whether xenon has a beneficial effect in preventing or reversing central sensitization after nerve injury or other chronic pain states.

AMPA receptors contribute to the acute spinal processing of both nociceptive and non-nociceptive inputs in the spinal cord [23,24]. Not only have they been implicated in analgesic action of anaesthetics [25,26], but accumulating evidence indicates that they contribute to synaptic plasticity associated with chronic pain states [27,28]. There is still an ongoing controversy whether xenon inhibits AMPA receptors. Different receptor composition probably underlies the variability in effect among tissues. Initial reports using hippocampal cultures [7,9] presumed lack of effect of xenon on AMPA receptors, because the effect of xenon on compound EPSCs closely mimicked the effect of a NMDA receptor antagonist. Another study using recombinant receptors [29] proposed that xenon induced inhibition of AMPA current is due to desensitization, since such inhibition was not observed when glutamate was applied briefly in high concentration to receptors containing GluR1 or GluR4 subunits. However, heteromeric GluR1/GluR2 receptors could not be tested with rapid pulses of glutamate in the above study. Since AMPA receptors formed by GluR1 and GluR2 subunits are thought to be predominant in the superficial dorsal horn [30], the sensitivity of AMPA receptors there could be different. Spinal cord slice-preparation allows recording of spontaneous and evoked synaptic events from neurons with native receptors and neurotransmitters and a relatively spared neuronal network, thereby eliminating unwanted artefacts such as questionable subunit composition and desensitization concerns. Our data from *in vitro *experiments clearly indicate that xenon inhibits AMPA receptors located on SG neurons receiving direct mono synaptic input from the primary afferent Aδ- and C-fibres, thus decreasing the amount of excitatory flow to the CNS.

These data were confirmed by *in vivo *experiments, in which xenon decreased the responses to innocuous and noxious stimuli. Moreover, the inhibitory effect of xenon did not change in the presence of APV, an NMDA receptor antagonist. However, adding CNQX resulted in almost complete vanishing of spontaneous and evoked EPSCs, suggesting that the above responses, recorded at holding potential of -70 mV, are predominantly AMPA receptor mediated.

The sensation for pinch is carried by Aδ- and C-fibres and touch could be relayed by Aβ-, Aδ- or C-fibres [31]. However, the monosynaptic input from Aβ-fibres is extremely rare in SG neurons under physiological conditions. Hypothesizing that the response to touch is predominantly conveyed by fine myelinated or unmyelinated fibres, the inhibition of the touch response by xenon would concomitantly inhibit the conduction of noxious responses since the same fibres convey the sensation of pain.

Taken together, our *in vitro *and *in vivo *data support the accumulating evidence from other studies using cortical cultures [10], amygdala slices [11] and behavioural tests on *C. elegans*[32] that AMPA receptors are also targets for xenon. It is difficult from our data to compare the sensitivity of the two types of ionotropic glutamate NMDA and AMPA receptors to xenon, but amygdala neurons [11] showed similar degree of inhibition of both receptors by several concentrations of xenon. Moreover, these receptors were similarly inhibited by xenon according to a freshly published study on SG and cortical neurons [33].

Since the rats for *in vivo *experiments were already anesthetized when xenon was applied, there is a possibility that urethane would affect the xenon action. Indeed, a modest potentiation of recombinant GABA and glycine receptors and modest inhibition of NMDA and AMPA receptors by urethane has been previously reported [34]. However, data from experiments with recombinant receptors sometimes fail to be confirmed by experiments using native receptors, like in the following study where urethane was shown not to affect the evoked Aδ- and C-fibre mediated EPSCs in spinal cord slice preparation [35]. Finally, the *in vivo *results in our study are in agreement with the results *in vitro*, where urethane was not used. Therefore it seems that urethane is unlikely to interfere with the effect of xenon.

There is an apparent difference in the extent of inhibition between evoked and miniature EPSCs. The purpose of investigation of miniature postsynaptic currents is to differentiate between pre- and post-synaptic site of action. The nature of the miniature events is different from that of evoked ones. Furthermore, the magnitude of drug effect could also be different. In addition, there is a detection threshold for the amplitude of mEPSCs, which implies the possibility that some events just above the threshold would become sub-threshold during xenon application leading to some underestimation of the degree of inhibition.

Many inhalational and venous anaesthetics potentiate inhibitory synaptic transmission, but we did not find a substantial effect of xenon on inhibitory synapses in the superficial dorsal horn. Xenon has been shown to increase the efficacy of GABA at recombinant receptors [36], but there are at least two striking differences other than receptor composition itself between our study and Hapfelmeyer's. We used 50% xenon vs. 100% and we used 10^-3 ^M GABA vs. 10^-5^-10^-7 ^M. Our results are consistent with most other reports, which agree on the negligible effect of xenon on GABA and glycine receptors [7,11,19,37].

Xenon was found to possess an equal or superior analgesic effect to nitrous oxide in MAC-equivalent concentrations in humans [16,17]. Although a quantitative comparison is beyond the scope of this work, we found that the 50% xenon used here and the 50% nitrous oxide used in our previous work [38] had comparable effects on glutamate receptors in SG neurons. Moreover, besides glutamate receptor inhibition in the dorsal horn, the descending noradrenergic system also participates in nitrous oxide-induced analgesia. That said, actions of xenon on higher centres and/or other targets like acetylcholine and serotonin receptors, two-pore domain potassium channels or plasma membrane Ca^2+^-adenosine triphosphatase may also contribute to xenon's analgesic effect.

## Conclusions

In conclusion, our data identifies NMDA and AMPA receptors in SG neurons as molecular targets of xenon, and suggests that the inhibition of these receptors underlies the anti-nociceptive action of xenon.

## Methods

### *In vitro *patch-clamp recordings

The methods used for the current experiment were similar to those used in our previous study [39] and were approved by the Animal Care and Use Committee at Niigata University Graduate School of Medical and Dental Sciences. Briefly, male Wistar rats (200-300 g) were anaesthetized with urethane (1.5 g/kg, i.p.). A dorsal laminectomy was performed and a lumbosacral segment of the spinal cord was removed. A 500-μm-thick transverse slice was cut sparing the left L4 dorsal root, on a DTK-1500 vibrating microslicer (Dosaka, Japan), and placed on a nylon mesh in the recording chamber. The slice was perfused with Krebs solution (10-15 ml/min) equilibrated with a 95% O_2_, 5% CO_2 _gas mixture at 36°C (See additional file 3). The composition of the Krebs solution was as follows (in mM): NaCl 117, KCl 3.6, CaCl_2 _2.5, MgCl_2 _1.2, NaH_2_PO_4 _1.2, NaHCO_3 _25 and D-glucose 11. Whole-cell patch-clamp recordings were obtained from SG neurons in voltage-clamp mode with patch pipettes pulled from thin-walled glass capillaries (1.5 mm o.d., World Precision Instruments) to have a tip resistance of 5-8 MO. The patch-pipette solution contained (in mM): Cs_2_SO_4 _110, CaCl_2 _0.5, MgCl_2 _2, EGTA 5, HEPES 5, tetraethyl ammonium (TEA) 5, ATP (Mg salt) 5. The SG was visually identified as a translucent band and the tip of the recording electrode was directed toward its centre.

Signals were amplified with an Axopatch 200B amplifier (Molecular Devices) and were filtered at 2 kHz and digitized at 5 kHz. All experiments were performed in voltage-clamp mode. Holding potential was set to -70 mV for recording exogenous AMPA current and miniature and evoked EPSC. Exogenous NMDA current was recorded at +40 mV. Exogenous GABA and glycine currents, as well as miniature and evoked IPSCs, were recorded at 0 mV.

Excitatory synaptic currents were evoked by dorsal root electrical stimulation, performed at relatively low intensity for Aδ-fibres (30-100 μA, 0.1 ms), and at relatively higher intensity and longer duration for C-fibres (200-700 μA, 0.5 ms). Differentiation of synaptic responses into Aδ- and C-fibres was based on a combination of response threshold and conduction velocities (5-8 m/s for Aδ-fibres; 0.5-1.0 m/s for C-fibres [38]). Evoked EPSCs that displayed a constant latency and lack of failures with high frequency stimulation (20 Hz for Aδ-fibres and 1 Hz for C-fibres) were classified as monosynaptic. Inhibitory synaptic responses were evoked by focal stimulation of SG interneurones with a monopolar silver wire electrode (diameter = 50 μm), insulated except for the tip and located within 100 μm of the recorded neuron [40].

### *In vivo *patch-clamp recordings

As previously described [41,42], male Wistar rats aged 7-8 weeks were anaesthetized, lumbar laminectomy was performed at the level of L4 or L5, and then the animal was placed in a stereotaxic apparatus. After removing the *dura mater *and cutting into the arachnoid membrane a window large enough to let pass a patch electrode, the surface of the spinal cord was irrigated with 95% O_2_, 5% CO_2 _equilibrated Krebs solution. Whole-cell patch-clamp recordings were performed as described in the *in vitro *section. The recorded neurons were identified as being in the SG based on their morphological features revealed by the depth of the neurons from the surface of the spinal cord and on their electrophysiological properties [41]. The mechanical stimuli used were pinches of skin folds with a toothed forceps and light touch of the surface of skin or the hairs in the ipsilateral hind limb. The stimuli were applied at the maximal response point of the respective receptive area and care was taken to stimulate the same point during the recording. The amplitude of the EPSCs during the stimulation were measured for a fixed interval according to the stimulus duration (typically 5-8 sec), then averaged and compared to the control values for each neuron. The area under the curve is a single value calculated in pCLAMP10 (Molecular Devices). The n values are the number of neurons.

### Drugs and data analysis

Xenon was applied by bubbling through the perfusing solution and was delivered through ultrathin polyethylene tubes. The solution was first saturated with the gas mixture for 30 min and then applied to the slice for at least 5 min prior to recordings. Xenon was mixed in a 1:1 ratio with a prefabricated gas containing 90% O_2_, 10% CO_2 _so that the composition of the gas mixture was: 50% Xe, 45% O_2_, and 5% CO_2_. In some experiments 50% nitrogen was used instead of xenon as a negative control. NMDA (50 μM), AMPA (10 μM), GABA (1 mM) and glycine (1 mM) were applied to the slice for 30 sec. In a subset of experiments tetrodotoxin (TTX, 1 μM) was used to isolate mEPSCs and mIPSCs. Drugs were purchased as follows: xenon from TG Showa (Tokyo, Japan), NMDA, AMPA, APV, CNQX, GABA, glycine from Sigma (USA) and TTX from Wako (Japan).

Data were analyzed using pCLAMP10 (Molecular Devices), GraphPad Prism 5 (GraphPad Software) and Mini-analysis 6.0.3 (Synaptosoft) software. Data are presented as means ± SEM. Because most of the data met the criteria for parametric tests, they were analyzed by Student's paired *t*-tests, unless otherwise stated. For detection of mEPSCs and mIPSCs, the amplitude threshold was defined as three times the noise level and the detected events were subsequently visually confirmed. The effect of xenon on the cumulative distribution of mEPSC and mIPSC amplitudes and inter-event intervals was analyzed using the Kolmogorov-Smirnov test. P < 0.05 was considered significant and is indicated by an asterisk in the figures.

## Competing interests

The authors declare that they have no competing interests.

## Authors' contributions

All authors read and approved the manuscript. SKG designed the study, performed the *in vitro *experiments and wrote the manuscript. HF performed the *in vivo *experiments. HB participated in the design of the study and performed the statistical analysis. TK conceived of the study, participated in the study design, and reviewed the manuscript.

## Supplementary Material

Additional file 1 **Xenon inhibited the responses to touch *in vivo *in the presence of APV**. Representative traces of response to touch (A) in presence of APV (50 μM; B), APV and xenon (C), and APV and CNQX (20 μM) (D). Stimulus duration is marked with a bar. Responses to touch in the presence of APV had similar amplitude (98 ± 1%, n = 3) and integrated area (97 ± 2%, n = 3) compared to control. Xenon inhibited the response to touch (amplitude (E): 64 ± 2%; area (F): 62 ± 2%, n = 3) even in the presence of APV. P < 0.05 is indicated by an asterisk. The touch response was CNQX sensitive (D, E, F). Click here for file

Additional file 2 **Xenon inhibited the responses to pinch *in vivo *in the presence of APV**. Representative traces of response to pinch (A) in the presence of APV (50 μM; B), APV and xenon (C), and APV and CNQX (20 μM) (D). Stimulus duration is marked with a bar. Responses to pinch in the presence of APV had similar amplitude (96 ± 5%, n = 4) and integrated area (97 ± 3%, n = 4) compared to control. Xenon inhibited the response to pinch (amplitude (E): 65 ± 3%; area (F): 61 ± 3%, n = 4) even in the presence of APV. P < 0.05 is indicated by an asterisk. The pinch response was also CNQX sensitive (D, E, F). Click here for file

Additional file 3 **Schematic representation of the experimental setting for *in vitro *recordings**. A spinal cord slice (scaled-up for clarity) is being continually perfused with artificial cerebrospinal liquid. Membrane currents are recorded from a lamina II neuron by perforating its cellular membrane with a recording pipette. Electrical stimulation of the dorsal root evokes postsynaptic currents, which after being amplified and digitalized, are recorded on a personal computer. Click here for file
