# Supplementary figures and images for: Xenon inhibits excitatory but not inhibitory transmission in rat spinal cord dorsal horn neurons
Source: Mol Pain. 2010 May 5;6:25. doi: 10.1186/1744-8069-6-25 (PMC2873505; doi:10.1186/1744-8069-6-25)

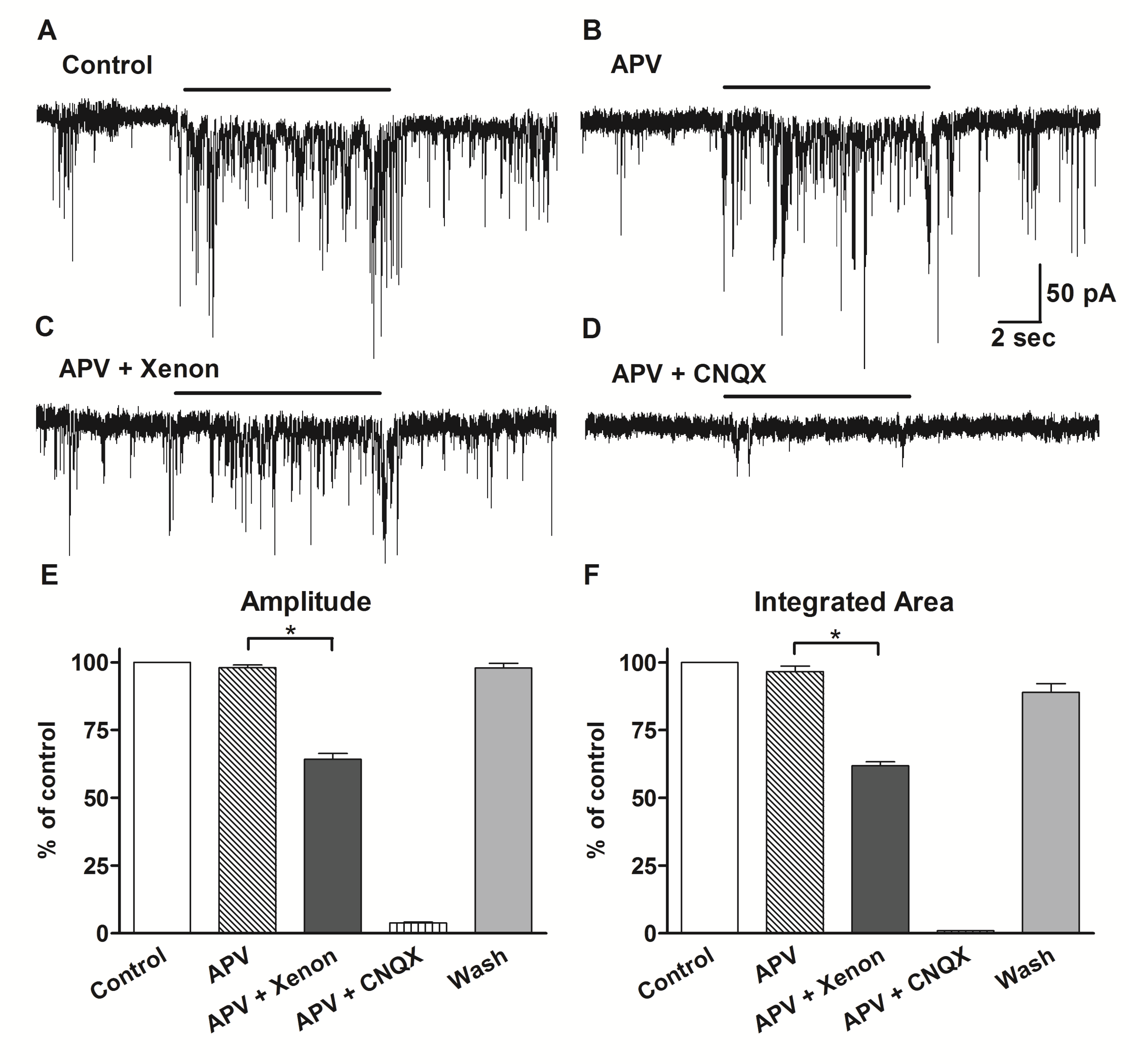

Supplement: Additional file 1 — Xenon inhibited the responses to touch in vivo in the presence of APV. Representative traces of response to touch (A) in presence of APV (50 μM; B), APV and xenon (C), and APV and CNQX (20 μM) (D). Stimulus duration is marked with a bar. Responses to touch in the presence of APV had similar amplitude (98 ± 1%, n = 3) and integrated area (97 ± 2%, n = 3) compared to control. Xenon inhibited the response to touch (amplitude (E): 64 ± 2%; area (F): 62 ± 2%, n = 3) even in the presence of APV. P < 0.05 is indicated by an asterisk. The touch response was CNQX sensitive (D, E, F). [file 1744-8069-6-25-S1.TIFF]

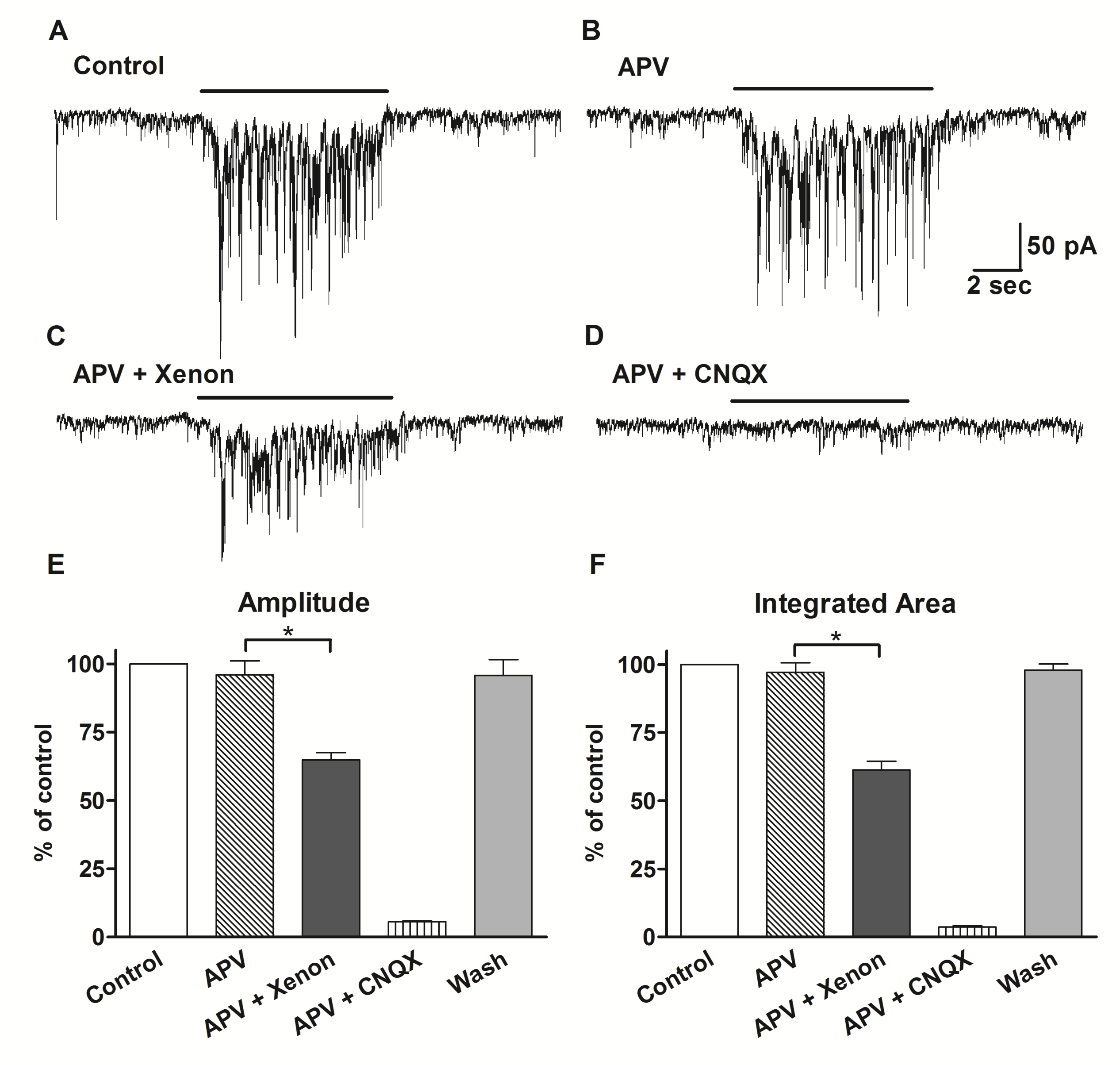

Supplement: Additional file 2 — Xenon inhibited the responses to pinch in vivo in the presence of APV. Representative traces of response to pinch (A) in the presence of APV (50 μM; B), APV and xenon (C), and APV and CNQX (20 μM) (D). Stimulus duration is marked with a bar. Responses to pinch in the presence of APV had similar amplitude (96 ± 5%, n = 4) and integrated area (97 ± 3%, n = 4) compared to control. Xenon inhibited the response to pinch (amplitude (E): 65 ± 3%; area (F): 61 ± 3%, n = 4) even in the presence of APV. P < 0.05 is indicated by an asterisk. The pinch response was also CNQX sensitive (D, E, F). [file 1744-8069-6-25-S2.TIFF]

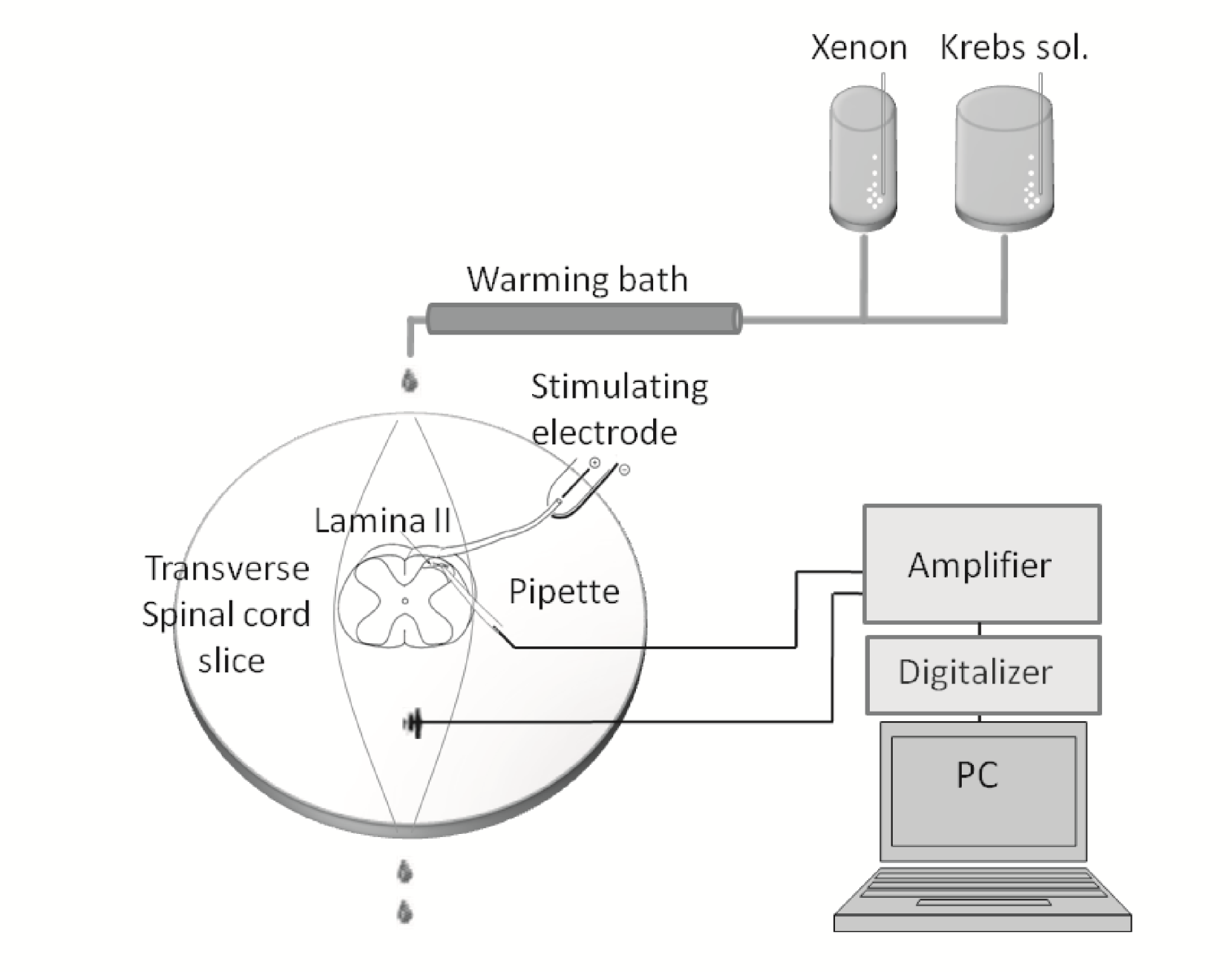

Supplement: Additional file 3 — Schematic representation of the experimental setting for in vitro recordings. A spinal cord slice (scaled-up for clarity) is being continually perfused with artificial cerebrospinal liquid. Membrane currents are recorded from a lamina II neuron by perforating its cellular membrane with a recording pipette. Electrical stimulation of the dorsal root evokes postsynaptic currents, which after being amplified and digitalized, are recorded on a personal computer. [file 1744-8069-6-25-S3.TIFF]
